# Analysis of the anti-tumor effect of cetuximab using protein kinetics and mouse xenograft models

**DOI:** 10.1186/1756-0500-4-140

**Published:** 2011-05-10

**Authors:** Teppei Matsuo, Satoshi S Nishizuka, Kazushige Ishida, Takeshi Iwaya, Miyuki Ikeda, Go Wakabayashi

**Affiliations:** 1 Molecular Therapeutics Laboratory, Department of Surgery, Iwate Medical University School of Medicine, 19-1 Uchimaru, Morioka 020-8505, Iwate Japan

## Abstract

**Background:**

The binding of EGFR and its ligands leads to autophosphorylation of receptor tyrosine kinase as well as subsequent activation of signal transduction pathways that are involved in regulating cellular proliferation, differentiation, and survival. An EGFR inhibitor, cetuximab binds to EGFR and consequently blocks a variety of cellular processes. *KRAS*/*BRAF *mutations are known to be associated with a low response rate to cetuximab. In the present study, to clarify the anti-tumor mechanisms of cetuximab, we evaluated the *KRAS*/*BRAF *status, phosphorylation level of the EGFR pathway, and the tumor suppression effect in vivo, using a human colon cancer cell line HT29, which exhibited the highest EGFR expression in response to the cetuximab therapy among the 6 colorectal cancer cell lines tested.

**Findings:**

The conventional growth suppression assay did not work efficiently with cetuximab. EGF, TGF-α, and IGF activated the EGFR/MAPK cell signaling pathway by initiating the phosphorylation of EGFR. Cetuximab partially inhibited the EGFR/MAPK pathway induced by EGF, TGF-α, and IGF. However, cetuximab exposure induced the EGFR, MEK, and ERK1/2 phosphorylation by itself. Mouse xenograft tumor growth was significantly inhibited by cetuximab and both cetuximab-treated and -untreated xenograft specimens exhibited phosphorylations of the EGFR pathway proteins.

**Conclusions:**

We have confirmed that cetuximab inhibited the EGFR/MAPK pathway and reduced tumor growth in the xenografts while the remaining tumor showed EGFR pathway activation. These results suggest that: ( i ) The effect of cetuximab in growth signaling is not sufficient to induce complete growth suppression in vitro; ( ii ) time-course monitoring may be necessary to evaluate the effect of cetuximab because EGFR signaling is transmitted in a minute order; and ( iii ) cetuximab treatment may have cells acquired resistant selectively survived in the heterogeneous cancer population.

## Background

The epidermal growth factor receptor (EGFR) is a transmembrane glycoprotein that constitutes one of four members of the erbB family of tyrosine kinase receptors [1]. EGFR regulates the key processes of cell biology, including proliferation, survival, and differentiation during development and tissue homeostasis [2]. One of the most common approaches to inhibit EGFR signaling in the anticancer therapeutic context has been to develop monoclonal antibodies against EGFR. The monoclonal antibody 225 (i.e. cetuximab) is one of several antibodies raised by inoculation of mice with A431 epidermoid carcinoma cells [3] and it was subsequently selected to generate a human mouse chimeric molecule for clinical development [4]. Cetuximab (marketed under the name Erbitux) is a chimeric (mouse/human) monoclonal antibody that inhibits the epidermal growth factor receptor (EGFR) [3]. It has been given by intravenous infusion to treat metastatic colorectal cancer and head and neck cancer [5].

It has been reported that cetuximab increases survival in patients with advanced CRC when administered in combination with irinotecan and fluorouracil [6]. Results from other clinical trials have also indicated that the benefit from the addition of cetuximab to first-line chemotherapy seems to be restricted to patients with the wild-type KRAS gene; with the best outcomes being observed in those with unmutated forms of both the KRAS and BRAF genes [6-8]. A detailed understanding of the mechanisms controlling cetuximab antitumor activity is necessary to optimize its therapeutic efficacy and to identify those patients who are likely to benefit from the treatment. Although KRAS and BRAF gene status has been considered as a meaningful biomarker, EGFR signaling can also be regulated by several ligands, receptors and cross-talk with other pathways. Hence, these gene mutations may contribute to individual tumors in varying degrees. Moreover, EGFR signaling could change in minutes order so it is crucial to monitor the dynamics of signal transduction to understand the intrinsic biological entity of tumor growth, in addition to the representative gene mutation status. To understand such signaling kinetics and tumor growth suppression in response to cetuximab administration, we used a colorectal cancer cell line, HT29, which expressed the highest EGFR level of 6 colorectal cancer cell lines tested. Here, we report the signaling alteration of the EGFR/MAPK pathway initiated by three types of growth factors in the presence/absence of cetuximab in vitro. The protein phosphorylation status of the xenograft tumor treated with cetuximab will also be discussed.

## Material and methods

### Colon cancer cell lines

Six human colorectal cancer (CRC) cell lines were used for molecular characterization. HT29 was obtained from the American Type Culture Collection. HCT116 was obtained from the U.S. National Cancer Institute and CW2, JHSK-rec, JHCOLO-YI, and TT1TKB were obtained from the RIKEN Cell Bank. HT29, CW2, and HCT116 were grown in RPMI 1640 supplemented with 10% fetal bovine serum. JHCOLO-YI and JHSK-rec were grown in DMEM/F12 with 10% FBS and 0.1mM non-essential amino acid and TT1TKB was grown in DMEM with 10% FBS. All cell lines were incubated at 37°C with 5% CO_2_.

### Reagents

The following 3 growth factors were used for growth signal stimulation: Epidermal Growth Factor (EGF, SIGMA-ALDRICH), Transforming Growth Factor-α (TGF-α, Pepro Tech House), and Insulin-like growth factor-I human (IGF, SIGMA-ALDRICH). Each growth factor was dissolved in the medium at the following concentrations: EGF, 100ng/ml; TGF-α, 50ng/ml; and IGF, 50ng/ml. Fluorouracil (5-FU, Kyowa Hakko Kirin), an anti-human EGFR monoclonal antibody, cetuximab (Erbitax, Bristol-Myers K.K.) were used in a serial dilution for growth suppression assays in vitro. When cetuximab was injected into nude mice (described details below), the dose was 10mg/kg.

### DNA sequencing

Human *KRAS *exon 2 (codon 12, 13) and *BRAF *sequencing were performed with genomic DNA subjected to PCR amplification with the following set of intronic primers: *KRAS *forward 5'-GGCCTGCTGAAAATGACTGA-3', reverse 5'-GTCCTGCACCAGTAATATGC-3' [9]; and *BRAF *exon 15 (codons 582-620) forward 5'-TGTTTTCCTTTACTTACTACACCTCA-3', reverse 5'-TCAGTGGAAAAATAGCCTCAA-3' [10]. The PCR products were then sequenced using the Big Dye Terminator V3.1 (Applied Biosystems) according to the protocol supplied by the manufacturer.

### Growth suppression assay

Cells (5,000 for cell/swell) were seeded on to a 96-well plate in an appropriate medium with FBS. Forty-eight hours later, the cells were treated with cetuximab with a 10-fold series of concentrations, and incubated for another 48 hours. Water-soluble tetrazolium salts were added to each well and incubated for 3-6 hours at 37°C (CCK-8, Dojindo). Absorbance was measured at 450 nm, with a reference filter at 520-540 nm, using a microplate reader.

### Western blot

Cells were seeded in a T-25 cell culture flask in RPMI1640 containing 10% FBS. After the cells reached 80% confluence, the medium was replaced with serum-free RPMI for 24 hours. Effects by serum starvation, such as induction of apoptosis, have been reported for various types of cells including gliomas, bladder and colon carcinomas [11-14]. There are several reports describing the effects of serum starvation in HT29 cells for 24 hours [15,16] and this procedure has been well accepted as a way of seeing the effect of cell growth signaling in general [17]. In the present study, we decided to set a 24 hours serum starvation sample as the control for the following EGFR signaling study. After the serum starvation was completed, the cells were stimulated with combinations of EGF 100ng/ml, TGF-α 50ng/ml, and IGF 50ng/ml, and incubated with cetuximab 300 μg/ml, resulting in a total of 7 conditions for 0, 1, 5, 10, 15 minutes. The cells were then washed with PBS and lysed with Pink Buffer containing 9 M urea (Sigma), 4% 3-[(3-cholamidopropyl)dimethylammonio]-1-propanesulfonate (CHAPS; Calbiochem), 2% pH 8.0-10.5 Pharmalyte (Amersham Pharmacia Biotech, Piscataway, NJ), and 65 mM DTT (Amersham Pharmacia Biotech) [18]. The full fraction of protein was resolved using NuPAGE^® ^4-12% Bis-Tris Gel electrophoresis under reducing conditions and transferred to a nitrocellulose membrane. The resulting membranes were blocked with 5% nonfat dried milk (cell signaling technology) in 0.1% Tween 20 (Bio-Rad) in PBS for 1 hour. Membranes were then incubated with primary antibodies; the dilution factors were all 1:1000 EGFR and p-EGFR(Tyr1045) (Cell Signaling Technology), p-MEK(ser218/ser222) (ECM Biosciences), p-ERK(Thr-202/Tyr-204) and pan-Actin (Thermo), overnight at 4°C. Next, the membranes were washed 3 times for 5 minutes in 0.1% Tween 20 in PBS, incubated with an HRP-conjugated secondary antibody for 1 hour at 4°C, and washed 3 times for 5 minutes in 0.1% Tween 20 in PBS. Chemiluminescence detection reagents (Thermo) were incubated with the membranes for 10 minutes, which was followed by film development. The signal intensities of phosphorylated EGFR, MEK, and ERK protein levels are expressed in an arbitrary unit (AU).

### Xenograft model

HT29 cells (1.0 × 10^7^) were injected subcutaneously into the right and left side of the back of 5 week old female athymic nude mice (CLEA japan: Central laboratory for experimental animals Japan). Tumor volume was calculated every 2-3 days with a caliper and using the following formula: Volume = length × width × height. After the tumors reached approximately 100mm^3^, either 40 μl of saline solution or 40 μl of cetuximab solution (5mg/ml) was intravenously administered to each mouse twice a week. Animals care was in accordance with our institution's guidelines. All animal experiments in this study were approved by the Iwate Medical University Ethical Committee for Animal Experiment Regulation, 22-007, Morioka, Japan.

### Immunohistochemistry

After euthanization of the mice, the tumors were excised and preserved in 10% formalin. Hematoxylin and eosin (HE) staining was performed using a standard technique. Immunohistochemical (IHC) staining procedures for total EGFR were as follows: Paraffin was depleted from a slide and incubated with pepsin solution for 20 min at 37°C. Tissue sections were covered with 3% H_2_O_2 _to block endogenous peroxidase for 10 min at RT (room temperature). Slides were incubated with anti-EGFR at 1:50 (NICHIREI Bioscience) for 60min at RT. After washing TBS (Tris-Buffered saline), the slides were incubated with a secondary antibody (EnVision™: DAKO JAPAN) for 30 min at RT. Tissue staining was visualized using a DAB (3, 3'-[[diaminobenzidine]]) substrate chromogen solution.

IHC staining for phosphorylated proteins was conducted as follows: After the same preprocessing of the anti-EGFR antibody as described above, tissue sections were blocked with protein block serum free to prevent nonspecific reactions for 10 min at RT. Slides were incubated with the primary antibody [pEGFR(1045) at 1:50, Cell Signaling Technology], [pERK (Thr202/Tyr204) Rabbit mAb at 1:300, Cell Signaling] Technology overnight at 4°C. The slides were then incubated with the secondary antibody (DAKO JAPAN) for 15 min at RT. Next, the slides were incubated with tyramide for 15 min. Tissue staining was visualized using a DAB substrate chromogen solution. The slides were then counterstained with hematoxylin, dehydrated, and mounted.

## Results

### Characterization of colorectal cancer cell lines

**Figure 1:**
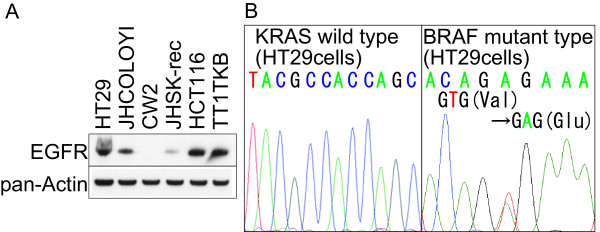
**Characterization of the colorectal cancer cell lines**. Protein expression of EGFR in colorectal cancer cell lines and sequence histograms of HT29, A, Expression of EGFR in HT29, CW2, HCT116, JHSK-rec, JHCOLO-YI and TT1TKB cells determined by Western blot. Pan-Actin was used as a loading control. B, Sequencing analysis of KRAS and the BRAF gene in HT29 cells.

Total EGFR protein expression was detected in HT29, JHCOLO-YI, JHSK-rec, HCT116, and TT1TKB by Western blot analysis. Three cell lines (HT29, HCT116, and TT1TKB) exhibited a relatively high level of protein expression, while one (JHCOLOYI) was intermediate, and 2 (CW2 and JHSK-rec) were very weak or showed no expression (Figure 1A). Sequencing analysis revealed that HT29, JHCOLO-YI, and CW2 were *KRAS *wild-type, whereas JHSK-rec, HCT116, and TT1TKB were *KRAS *mutant-type; JHCOLO-YI, JHSK-rec, HCT116, and TT1TKB were *BRAF *wild-type, whereas HT29 was *BRAF *mutant-type (Figure 1B) (Table 1).

**Table 1:** Gene mutation status and EGFR expression of colon cancer cell lines

Cell lines	EGFR	KRAS	BRAF
HT29	+++	wt	mt
JHCOLO-YI	++	wt	wt
CW2	-	wt	wt
JHSK-rec	++	mt	wt
HCT116	++	mt	wt
TT1TKB	++	mt	wt

Wt, wild type; mt, mutant type; +++, very strong positive; ++, strong positive; +, positive; -, negative. Level of EGFR is determined by Western blotting

### Growth suppression assay with cetuximab

**Figure 2:**
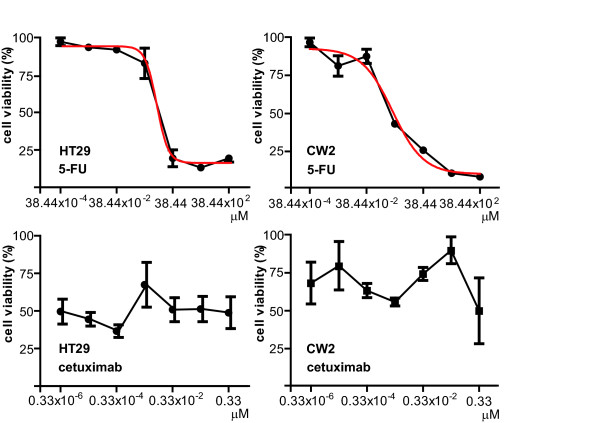
**Growth suppression assay for in vitro antitumor activity of cetuximab in CRC cell lines**. Growth suppression curves resulting from either 5-FU or cetuximab dose-dependent administrations for 48h are shown. The error bar shows mean ± SEM (standard error of the mean) of each concentration data point. The vertical axis represents the cell viability, and the horizontal axis represents the drug concentration.

To determine the effects of cetuximab on cell growth in vitro, we performed a growth inhibitory assay with 6 CRC cell lines. A conventional chemosensitivity test using 5-FU or cetuximab was performed to identify if a growth inhibitory effect could be seen in a dose-dependent manner. As predicted, 5-FU exhibited a growth suppression curve with a dose-dependent manner in 4 out of 6 cell lines, whereas growth was not inhibited by cetuximab in any of the 6 cell lines (Figure 2).

### Signal transduction induced by growth factors and cetuximab

**Figure 3:**
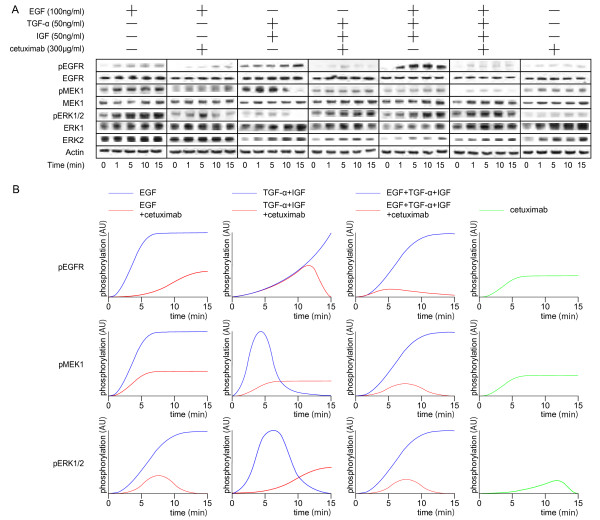
**Kinetics of proteins involved in EGFR/MAPK signaling with cetuximab in HT29 cells**. A, HT29 cells were treated with serum-free RPMI for 24 hours, followed by the addition of EGF 100ng/ml, TGF-α 50ng/ml, IGF 50ng/ml, and cetuximab 300ng/ml, resulting in a total of 7 conditions for 0, 1, 5, 10, 15 minutes before collections of protein lysate. The phosphorylation of EGFR, MEK, and ERK was visualized using Western blot analysis. Actin was used as a loading control. B, A Western blot band was plotted in an arbitrary unit showing the time course.

All growth factors used in the present study activated EGFR/MAPK signaling (Figure 3) and TGF-α and IGF induced phosphorylation of downstream signaling more quickly than EGF did. While EGF induced sustained phosphorylation, a cocktail of TGF-α and IGF induced either exponential or temporal phosphorylation. We next observed for how cetuximab alters the phosphorylation level and whether it induces dephosphorylation in the presence of the above growth factors. As expected, the level of growth factor induced phosphorelation is decreased in the presence of cetuximab. Since all three growth factors have an affinity for EGFR, it makes sense that cetuximab reduces the phosphorylation levels that they induce; however, cetuximab does not seem to inhibit any of the phosphorylation levels completely. Interestingly, cetuximab itself exhibited the induction of phosphorylation in the absence of growth factors at multiple levels of the EGFR pathway.

### Inhibition of tumor growth in the mouse xenograft

**Figure 4:**
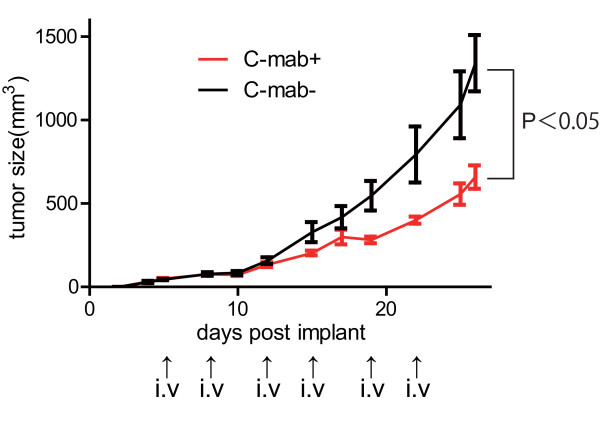
**In vivo tumor suppression by cetuximab**. Growth suppression of the HT29 mouse xenograft is present. HT29 xenografts were either untreated or treated with 200 μg of cetuximab per mouse twice a week. The tumor volume was measured every 2-3 days, and all mice were sacrificed after 26 days of treatment. Error bars represent the standard error of the mean. We compared the tumor volume of treated and untreated xenografts using the student t-test (*p *< 0.05).

**Figure 5:**
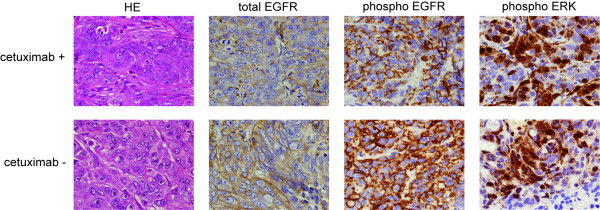
**Immunohistochemistry of xenograft tumors**. HT29 mouse xenograft tumors were removed and stained after cetuximab treatment. Tumor sections were stained with HE, anti-EGFR, anti-pEGFR(1045), anti-pERK(Thr202/Tyr204). Images are from cetuximab-treated (top) and -untreated (bottom).

Treatment with 6 injection of cetuximab for 26 days resulted in a significant suppression of tumor growth (P < 0.05) (Figure 4). To evaluate whether there were any differences in the EGFR/MAPK signaling between cetuximab treatment (+) and (-) in vivo, we measured the total EGFR, phosphor-EGFR, and phosphor-ERK staining in the xenograft tumor sections. There were no visible differences between cetuximab (+) and cetuximab (-) tumors in terms of cell/tissue structure, and no necrotic or hyperinflammatory features were found. Both EGFR and phosphor-EGFR were stained in the cell membranes although the degree of staining was not homogeneous within the tumor. Phosphor-ERK nuclear staining was seen in substantial parts of the tumors. Interestingly, phosphor-ERK positive cells were occasionally found next to or in the negative cells (Figure 5).

## Discussion

Expression of the EGFR protein was supposed to be the key molecule for cetuximab therapy indication. However, recent studies have indicated that EGFR IHC does not predict the need for cetuximab treatment. Subsequently analysis of the *KRAS *mutation has been widely accepted as giving a better prognostic value than EGFR [7,8,19]. In fact, our examination revealed that EGFR expression did not correlate with *KRAS *and *BRAF *status. HT29 had the *KRAS *wild-type with *BRAF *mutation, and no *BRAF *mutations were found in other cell lines with the mutated *KRAS *genotype. This is in agreement with previously published observations by other authors that mutations in *KRAS *and *BRAF *are mutually exclusive [20,21]. We chose HT29, which has the conventional criteria for cetuximab therapy (i.e. EGFR-positive and *KRAS *wild-type) to see how EGFR signal transduction was altered by the signal intervention with cetuximab.

In addition, we used a growth inhibitory assay to examine the cytotoxicity of cetuximab to clarify if a cell-based assay can be used in therapeutic decision making. Surprisingly, cetuximab did not suppress cell growth in vitro in any of the 6 cell lines, including HT29. A possible explanation for this is that antibody-class anticancer agents including cetuximab do not seem to be as cytotoxic as other conventional classes of anticancer drugs, which may require a host-dependent cytotoxic mechanism (i.e., immune system) [22]. Hence, the conventional growth suppression assay may not work efficiently with the antibody class drugs, at least cetuximab.

HT29 is an EGFR-positive, *KRAS *wild-type and *BRAF *mutant-type cell line. In practice, it is considered that EGFR-positive as well as *KRAS *wild-type colorectal cancer patients have satisfied criteria for cetuximab therapy. It has also been speculated that colorectal cancer patients with the *BRAF *mutant type may be considered to be a minor 'non-effective' group for cetuximab therapy as determined by clinical trials [6-8]. However, when it comes to individual patients, the grouping may not always be fully indicative. In fact, "the effective rate of *KRAS *mutant" and "non-response rate with the *KRAS *wild type" are 6.7% and 64.2%, respectively [7]. Although these rates cannot be ignored they may not be of concern in practice because the gene status is currently almost the exclusive recommended criterion. In addition to showing the molecular mechanisms of tumor growth suppression by cetuximab, our results provide an example showing that it is worthwhile to consider not only the gene status but also the signaling pathway involved in the therapy.

**Figure 6:**
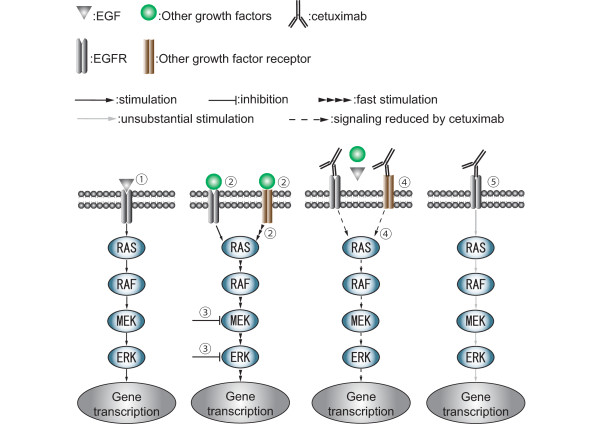
**Possible EGFR signaling pathways affected by cetuximab**. ➀EGF activates EGFR/MAPK signaling. ➁ Phosphorylation of downstream signaling is induced more quickly by TGF-α and IGF than EGF. ➂Potential negative-feedback from downstream signals. ➃Cetuximab reduces the phosphorylation level induced by the three growth factors. ➄Cetuximab binding to the receptor induces the receptor dimerization resulting in protein phosphorylation of downstream proteins. However, inhibition of the receptor internalization leads to the suppression of protein phosphorylation in downstream proteins (as a consequence, in the presence of cetuximab, the level of protein phosphorylation does exist but at a very low level).

All growth factors used in the present study activated EGFR/MAPK signaling and phosphorylation of downstream signaling was induced more quickly by TGF-α and IGF than EGF. This suggests that there is a cross-talk between EGFR/MAPK signaling and growth factor induced signaling other than EGF. While EGF induced sustained phosphorylation, a cocktail of TGF-α and IGF induced either exponential or temporal phosphorylation, suggesting that there is a negative-feedback circuit, which regulates the level of phosphorylation at the MEK/ERK level. Interestingly, cetuximab itself exhibited the induction of phosphorylation in the absence of growth factors at multiple levels of the EGFR pathway (Figure 3). In fact, it has been reported that cetuximab itself induced phosphorylation of EGFR at several tyrosine phosphorylation sites as a result of receptor dimerization and activation of the receptor tyrosine kinase [23-25]. On the other hand, direct occlusion of the ligand-binding site is the primary mechanism of inhibition by cetuximab [3,26]. Moreover, cetuximab-EGFR complexes are not removed from the plasma membrane, in contrast to the rapid receptor turnover induced by EGF alone [23,27,28]. The ligand-EGFR complex is rapidly internalized and then either recycled back to the cell surface or proteolytically degraded. The internalized EGFR interacts with various signaling proteins that are important for sustained activation of the major signaling pathways mediated by ERK [23,27,28]. Taken together, the previous reports and our present results indicate that cetuximab leads to receptor dimerization that results in EGFR signal activation to some degree; but the dimerization is a less substantial signal inhibitor than the internalization of the ligand-EGFR complex (Figure 6).

Other Investigators have hypothesized the mechanisms of action of cetuximab as follows: (i) Inhibits internalization of EGFR resulting in a lack of downstream phosphorylation [23]; (ii) Induces apoptosis by starving growth signals [3,23]; (iii) causes an immunological response by provoking ADCC [22]; and (iv) fills up the EGF binding site preventing EGF from binding to its receptor [3].

To measure the result of such mechanisms of action, reflecting the drug's potency, we used a widely available method to measure cell growth using a water-soluble tetrasolium salt, WST-8, a substrate of formazan. This presumably reflects the number of cells in a microplate well (i.e. CCK-8 assay). Hence, the assay system reactions are maintained as long as cells generate energy from biochemical reactions that are present such as glycolysis even if their growth is somewhat suppressed. In the present study, we found no evidence for clear cetuximab dose-dependent growth suppression with the assay (Figure 2) in contrast to other cytotoxic drugs such as 5-FU, which showed clear logistic growth suppression curves. However, we found visible suppression of protein phosphorylation in the EGFR signal transduction pathways suggesting that cetuximab affects the signal transduction in the surviving cells that seem to be unaffected at the cellular level. This may imply that it is difficult to see the growth suppression from cetuximab alone in the short term, for example when using a 48h cytotoxic assay. Based on previous reports and our present findings, it has been speculated that cetuximab may have a minimal direct cytotoxic effect by itself although an inhibitory effect of cetuximab on signal transduction in protein phosphorylation can be seen. The discrepancy between the growth suppression assay and the in vivo growth suppression effect of cetuximab may suggest that rapid growth suppression by cetuximab requires an extracellular effect.

The result of histological heterogeneous staining suggests that phosphorylation of the EGFR pathway components do not occur in a synchronized manner within the tumor. The fact that phosphor-EGFR positive cells are present in the tumor that has responded to cetuximab, also implies that cells which have acquired cetuximab may have selectively survived in response to the treatment.

## Conclusions

Although KRAS gene mutation status is clinically useful for therapeutic decision making, the molecular effect of targeted molecules and the downstream pathways have not yet been fully elucidated. In the present study, we assessed the effect of cetuximab in tumor growth at 3 different levels including the protein and, cell levels, and in vivo, with gene status and EGFR expression information. At the protein level, EGFR signaling was effectively inhibited as a consequence of cetuximab exposure, whereas growth suppression assay did not show a significant effect probably due to the lack of direct cytotoxicity. However, CRC cell line xenografts followed by cetuximab administration to nude mice exhibited significant growth suppression. The phosphorylation status of the cetuximab target pathway did not visibly differ between cetuximab-treated and -untreated tumors removed from the mouse. These results suggest that the antitumor effect of cetuximab cannot be predicted by conventional growth suppression assay alone. Examination of the phosphorylation level seems to be informative, but the level does not synchronize in tumor cells probably due to the level change occurring in minutes order in a heterogeneous cell population. Although the set of assays is labor intensive and time consuming, it may compensate for the missing part in the current decision making process for selecting cetuximab therapy, leading to the establishment of new criteria for individual patients.

## List of abbreviations used

CHAPS: 3-[(3-cholamidopropyl)dimethylammonio]-1-propanesulfonate; CRC: colorectal cancer 3, DAB: 3, 3'-[[diaminobenzidine]]; DMEM: dubecco's modified eagle medium; DMEM/F12: dubecco's modified eagle medium nutrient mixture F-12; DNA: deoxyribonucleic acid; DTT: dithiothreitol; EGF: epidermal growth factor; EGFR: epidermal growth factor receptor; ERK: extracellular signal-regulated kinase; FBS: fetal bovine serum; HE: Hematoxylin and eosin; IGF: insulin-like growth factor; IHC: Immunohistochemical; MAPK: mitgen-activated protein kinase; MEK: MAPK/ERK kinase; PBS: Phosphate Buffered Saline; PCR: polymelase chain reaction; RPMI: Roswell Park Memorial Institute medium; RT: room temperature; TBS: tris-buffered saline; TGF-α: transforming growth factor-α

## Competing interests

The authors declare that they have no competing interests.

## Authors' contributions

TM, SSN and GW conceived the study. TM, SSN, TI and GW were responsible for the experimental design. TM, SSN, KI, and MI performed experiments and helped in the analysis and interpretation. TM and SSN prepared the manuscript. All authors read and approved the final version of the manuscript.
